# 

**Published:** 1924-11

**Authors:** 


### RECENT APPOINTMENTS.

### Medical Officers, &c.

Allen, Leslie Paice ; School Dentist, Hertfordshire.

Atkinson, J. H. ; Medical Officer, Out-Patients' Department, National Hospital for Diseases of the Heart, W. 1.

Balmain, A. R., M.R.C.S., L.R.C.P., Assistant Medical Officer and Assistant School Medical Officer, Colchester ; Assistant Medical Officer, Birkenhead.

Carroll, F. R., M.R.C.S., L.R.C.P. ; Medical Officer of Health, Caterham.

Collins, F. Garland, M.R.C.S., L.R.C.P., D.P.H., Tuberculosis Officer, West Ham; Medical Officer of Health and Chief School Medical Officer, West Ham.

Cotton, T. F., M.D., M.R.C.P. ; Physician to Out-Patients, National Hospital for Diseases of the Heart.

Davey, E. V., M.B., B.Ch. ; Assistant Physician and Pathologist, Royal Infirmary, Gloucester.

Dowling, G. B., M.D., M.R.C.P., Physician to Skin Department, Miller General Hospital.

Fisher, T. N., M.B., Ch.B. ; Resident Surgical Officer, Pendlebury Hospital.

Griffiths, J. Vernon ; Medical Officer, General Dispensary, Birmingham.

Gross, Malcolm, M.R.C.S., L.R.C.P., M.B., B.S., D.P.H., Assistant Medical Officer, West Suffolk ; Assistant School Medical Inspector, Staffordshire.

Jardine, John, O.B.E., M.D., School Medical Officer and Assistant Medical Officer of Health, Peebles and Midlothian ; Medical Officer and Inspector of Physical Training, Scottish Education Department.

Jowell, A. H., M.R.C.S., L.R.C.P. ; Assistant Resident Medical Officer, Nottingham.

Low, E. B., M.B., Ch.B. ; Assistant Resident Medical Officer, Nottingham.

Lytle, W. J., M.B., F.R.C.S., Fulham Infirmary ; Resident Surgical Officer, Royal Hospital, Sheffield.

MacInnes, John, M.B., Asst. Medical Officer, Mental Hospital, Bexley ; Senior Assistant Medical Officer, City Mental Hospital, Hull.

Massen, A. A., M.D., B.Ch., Assistant-Medical Officer of Health, Liverpool ; Medical Officer of Health, Liverpool.

Maturin, Francis Henry, M.R.C.S., L.R.C.P. ; Medical Officer of Health, Lymington.

Miller, C. H., M.D. ; Honorary Assistant Physician, Royal Devon and Exeter Hospital.

Roxburgh, Anne May, M.B., Ch.B., D.P.H., School Medical Inspector and Deputy Medical Officer of Health, Scarborough.

Sellars, J. A., M.B., Ch.B. ; Honorary Assistant Physician, Royal Infirmary, Blackburn.

Snell, N. W., M.R.C.S., L.R.C.P., House Physician, King's College Hospital ; Assistant Medical Officer, St. Marylebone Hospital.

Thompson, Albert H., M.B., B.Ch., Junior Assistant Medical Officer, 2nd County Mental Hospital, Barnwood ; Junior Assistant Medical Officer, County Mental Hospital, Northampton.

Young, W. F., M.B., Ch.B., D.P.H., Assistant School Medical Officer, Liverpool ; Assistant School Medical Inspector, Staffordshire.

### Public Health and Hospital Appointments.

Anderson, M. W. ; Health Visitor, Leeds.

Arnold, E. E. ; Matron, Eye Hospital, Bristol.

Beswick, J. ; Health Visitor, Leeds.

Black, Jean M. G. ; Matron, City Maternity Hospital, York.

Bloor, Margaret ; School Nurse, Barnsley.

Campbell, A. G., A.R.R.C. ; Matron, Hamilton House Convalescent Home, Seaford.

Cooke, Jessie Edith ; Matron, Fulwood and Longridge Joint Hospital, Preston.

Cooper, Ida H. ; School Nurse, Barnsley.

Erskine, C. M. ; Matron, City Mental Hospital, Hull.

Gregg, E. J., Assistant Secretary, Queen's Hospital, Birmingham ; Secretary, West Kent General Hospital, Maidstone.

Hopkins, Rose ; Health Visitor, Gillingham.

Howell, M. C. ; Matron, Westbury Cottage Hospital, Wilts.

Jennings, Ivy Hope ; School Nurse and Health Visitor, Walthamstow Education Committee.

Landray, D. A. E. ; Health Visitor, Poplar.

Laycock, D. M. ; Superintendent, Queen Victoria's Jubilee Institute, Manchester (Ardwick).

McArdle, P. T., M.B., B.Ch., R.U.I. ; Master, National Maternity Hospital, Dublin.

Morgan, G. M. ; Health Visitor and School Nurse, Worcestershire.

Murchison, Dolann, Acting Assistant Matron, Royal Hospital for Sick Children, Glasgow ; Matron, Hartlepool Hospital.

Pearce, Annie ; Health Visitor, Lincolnshire Nursing Association.

Perrett, Florence M. ; Health Visitor, Wiltshire.

Reynolds, Ida ; School Nurse, Rotherham.

Rutherford, Eleanor F. ; Matron, Cottage Hospital, Morpeth.

Thompson, E. M. ; Matron, West Suffolk General Hospital.

Whitelaw, A. D., M.B., B.Ch., D.P.H. ; Temporary Assistant School Medical Officer, Norwich.

Wilks, E. V. ; Assistant Matron, General Infirmary, Bury.

### ANSWERS TO CORRESPONDENTS.

MARGERY.—There are quite a number of books on the subject. Probably Coué's book, "My Method, including American Impressions," is what you mean. It is published by Heinemann.

W.—The address of the Institute of Hospital Almoners is Denison House, Vauxhall Bridge Road, S.W.

